# Lightly Cooked Broccoli Is as Effective as Raw Broccoli in Mitigating Dextran Sulfate Sodium-Induced Colitis in Mice

**DOI:** 10.3390/nu10060748

**Published:** 2018-06-08

**Authors:** Yanling Wang, Elizabeth H. Jeffery, Michael J. Miller, Matthew A. Wallig, Yuanfeng Wu

**Affiliations:** 1 Department of Food Science and Human Nutrition, University of Illinois, 905 S. Goodwin Ave., Urbana, IL 61801, USA; ywang436@illinois.edu (Y.W.); mille216@illinois.edu (M.J.M.); 2 Department of Pathobiology, University of Illinois, 2001 S. Lincoln., Urbana, IL 61802, USA; mawallig@illinois.edu; 3 School of Biological and Chemical Engineering, Zhejiang University of Science and Technology, Liuhe Road 318, Hangzhou 310023, China; wuyuanfeng@zju.edu.cn

**Keywords:** lightly cooked broccoli, dextran sulfate sodium, gut barrier, IL-6 trans-signaling pathway

## Abstract

Dietary broccoli is anti-inflammatory. Past studies have typically investigated raw broccoli, even though most consumers prefer cooked broccoli, where the plant myrosinase is inactivated by heat, resulting in failure of formation of the anti-inflammatory bioactive compound sulforaphane (SF). This study compareed efficacy of lightly cooked broccoli (CB) containing greatly diminished myrosinase activity, with raw broccoli (RB), in mitigating colitis in dextran sulfate sodium (DSS)-treated mice. Male C57BL/6 mice were fed for two weeks on a 10% RB, 10% CB or control diet, all based on the AIN-93M diet. Half (*n* = 9) of each group received drinking water, half received 2.5% DSS in water for one week, starting from Day 7 of the diet. Even with far less plant myrosinase activity, CB was essentially as effective as RB in lessening damage by DSS, evidenced by decreased disease activity index, attenuated colon length shrinkage, less endotoxin (lipopolysaccharide) leakage into blood, and less severe colon lesions as assessed by histopathology. mRNA expression of pro-inflammatory cytokines indicated that broccoli anti-inflammatory action may be through inhibition of the IL-6 trans-signaling pathway, as evidenced by reversal of the DSS-increased expression of IL-6, CCR2 and vascular cell adhesion molecule 1 (VCAM-1).

## 1. Introduction

Accumulating research frequently suggests a role for chronic inflammation as a key mechanism within a myriad of life-threatening diseases, including cardiovascular disease (CVD), cancer, diabetes, and obesity. Among these is inflammatory bowel disease (IBD), which is associated with severe chronic inflammation in the gut. This is a common disease, affecting over 1% of US adults (~3 million in 2015) [1]. Not only is the incidence of IBD increasing [1], but untreated IBD can progress to colon cancer.

Many research articles suggest that sulforaphane (SF) from broccoli, one of the most studied phytochemicals, is among several key anti-inflammatory dietary components that may benefit those suffering from such diseases [2,3,4,5]. In the dextran sulfate sodium (DSS) mouse model, which is a commonly used animal model for IBD, SF has been found to attenuate DSS-induced colitis, evidenced by a decreased disease activity index (DAI), minimized weight loss, reduced loss of colon length, and less monocyte infiltration [2]. Moreover, a molecular level investigation confirmed that expression of the pro-inflammatory cytokine IL-6 was decreased by SF in DSS-treated mice, and that the expression of several Nrf2 target genes was increased. This suggests that the anti-inflammatory bioactivity of SF might be mediated through the Nrf2 pathway [2]. In recent years, not only SF, but also whole broccoli has shown promising anti-inflammatory activity. Using the mdr1a^-/-^IBD mouse model, a diet containing 10% fresh broccoli was shown to alter the gut microbiota and attenuate colitis, evidenced by an increase in the colon crypt length and in the number of goblet cells present in the colonic epithelium [6]. In contrast, in an AOM/DSS model, a glucosinolate-enriched fresh broccoli diet was reported to induce Nrf2 target genes effectively while failing to protect the host from colitis or carcinogenesis [7].

In these studies, however, only fresh broccoli or SF was investigated. It has been widely acknowledged that the cooking conditions for broccoli greatly impact its efficacy. The hydrolysis from glucoraphanin (GRP), the parent compound of SF, is mediated by a heat-sensitive enzyme in broccoli, myrosinase (EC 3.2.1.147). In fully cooked broccoli, the myrosinase is likely to be inactivated by heat, resulting in failure to produce bioactive SF. Researchers have shown that the gut microbiome is also capable of hydrolyzing glucoraphanin (GRP) within dietary broccoli to release bioactive SF, although the yield is quite low [8]. Compared to fully cooked broccoli, the study of lightly cooked broccoli has become appealing to many researchers [9,10] as it provides the taste/flavor that consumers find more acceptable than raw broccoli, while still retaining some myrosinase activity. In the present study, we compared the efficacy of raw broccoli and lightly cooked broccoli in terms of protecting mice from DSS-induced colitis, as a model of IBD.

Although the etiology of IBD is unknown, it has been proposed that it is related to a “leaky gut”: increased gut permeability to luminal pathogens and their by-products, including endotoxin [11,12]. One common method to determine gut barrier permeability is to assess plasma lipopolysaccharide (LPS) concentration; LPS is a large molecule in the outer membrane of gram-negative bacteria, not able to pass across the intact gut barrier [13]. Another way is to determine the urinary excretion of orally ingested sucralose [13], a non-digestible sweetener that similarly cannot penetrate the intact gut barrier. However, if the integrity of the gut barrier is compromised and gut leakage occurs, LPS and sucralose enter the circulatory system. Therefore, in the present study, plasma LPS and urinary sucralose were used as indicators of gut barrier function. In addition, it is reported that the breach of the gut barrier, through destruction of colonocyte tight junction proteins, might be one of the mechanisms of IBD. Therefore, both mRNA expression of selected tight junction proteins and protein expression of two barrier proteins, ZO-1 and Claudin-1, were also assessed. Moreover, mRNA expression of selected pro-inflammatory cytokines was investigated to identify any inflammatory role within the molecular mechanism of broccoli protection of the host from DSS induced colitis. 

In this study, our results clearly show that even with far less myrosinase activity, lightly cooked broccoli is essentially as effective as raw broccoli in suppressing damage by DSS, for most of the endpoints. In addition, we propose here that broccoli protection against DSS-induced colitis may be through halting the transition from acute to chronic inflammation through SF-Nrf2 inhibition of the IL-6 trans-signaling pathway.

## 2. Materials and Methods

### 2.1. Diet Preparation

AIN-93M diet ingredients were purchased from Harlan-Teklad Laboratories (Madison, WI, USA). Fresh broccoli was purchased from a local grocery market. Broccoli heads were cut less than 2 inches from the crown and divided into florets of ~2-inch pieces. Half of the broccoli was lightly cooked by microwave for 3 min and cooled on ice, as described in our previous study [10]. Both broccoli preparations were then freeze dried and ground into powder, using a coffee grinder. Raw or lightly cooked broccoli powder (10% by weight) was incorporated into the raw broccoli diet (RB) or lightly cooked broccoli diet (CB), respectively. RB and CB were balanced to the AIN93M diet (CON) for micro- and macro-nutrients, including fiber (Table 1). Based on USDA nutrient database, there is approximately 2.3 g fiber/100 g fresh broccoli; therefore, we added 2.6 g cellulose to each broccoli diet to balance the 5 g cellulose in the control diets.

### 2.2. Animal Use and Experimental Design

Eight- to ten-week-old male C57BL/6 mice (*n* = 54, BW 20–25 g) were maintained under a 12-h light/dark cycle at 22 °C and 60% humidity. All animal care followed the protocol approved by the Institutional Animal Care and Use Committee at the University of Illinois, Urbana-Champaign. After four days of acclimation to the AIN-93M diet, mice were divided into three groups and provided with AIN-93M diet (CON), RB or CB for 14 days, until the end of the study. Diet was provided ad libitum and replaced fresh daily. On the seventh day of diet treatment, mice from each diet treatment group were divided randomly into two halves (*n* = 9). One half of the mice from each group received normal tap water (Water) and the other half received 2.5% DSS (40–50 kDa, Affymetrix, Santa Clara, CA, USA) dissolved in tap water from Day 7 to Day 14, the completion of the study. The six treatment groups are abbreviated as Water/CON, Water/RB, Water/CB, DSS/CON, DSS/RB, and DSS/CB.

### 2.3. Diet Analysis

Myrosinase activity in the broccoli diets was measured as SF formation in vitro, based on our previously published protocol [14]. Briefly, 400 mg freeze-dried RB or CB diet was incubated in 1.5 mL of water for different time periods (up to 8 h) with thorough vortexing in the middle of the incubation period. Samples were then centrifuge at 4 °C for 3 min. After centrifugation, an aliquot of supernatant (0.5 mL) was spiked with an internal standard of benzyl isothiocyanate, then immediately extracted into dichloromethane (DCM, 0.5 mL), centrifuged for 3 min at 16,000× *g* and analyzed for SF formation, by GC-FID. The GC program was as follows: briefly, 1 µL of DCM extract was injected, using an Agilent model 7683B series autosampler, onto an Agilent 6890N gas chromatography system equipped with a single flame ionization detector (Agilent Technologies, Santa Clara, CA, USA). Samples were separated using a 30 m × 0.32 mm J&W HP-5 capillary column (Agilent Technologies). After an initial hold at 40 °C for 2 min, the oven temperature was increased by 10 °C/min to 260 °C and held for 10 min. Injector temperature was 200 °C; detector temperature was 280 °C. Helium carrier gas flow rate was 25 mL/min. Data were quantified against a standard curve of SF (LKT Laboratories, St. Paul, MN, USA).

### 2.4. Tissue Collection

Mice were anesthetized using ketamine/xylazine (87 mg/mL and 13 mg/mL, respectively, at 0.1 mL/100 g BW). After blood was taken by cardiac puncture into EDTA-coated vessels, mice were killed by cervical dislocation. The colon, from immediately distal to the cecum to the anus (including the rectum; ~3–6 cm) was removed and immediately flushed with ice-cold PBS. After the length was measured, 16% of the length immediately distal to the midpoint was removed and fixed in 10% neutral buffered formalin. After ~24 h, sections were transferred into 80% ethanol for slide preparation by the Veterinary Diagnostic Laboratory (University of Illinois, Urbana, IL, USA). The remaining colon was scraped to collect mucosa for Western blot (stored at −80 °C) and qPCR analysis (placed into RNALater and frozen).

### 2.5. Disease Activity Index

Mice were monitored each day for observable changes in colitis symptoms. The disease activity index (DAI) was calculated by daily summing the colitis clinical scores, including percent weight loss, stool formation and presence of stool blood; scoring was adapted from reported protocol [15] as follows: (i) body weight loss: 0, none; 1, 1–5%; 2, 5–10%; 3, >10%; (ii) stool formation: 0, normal pellet; 1, loose stool (mild); 2, loose stool (moderate); 3, watery diarrhea; and (iii) fecal bleeding: 0, negative; 1, blood in stool (mild); 2, blood in stool (moderate); 3, gross bleeding. Mice were given a score of 0–3 for each of the three categories and the sum of scores was used as a measure of the degree of colitis as follows: 1–4: Mild colitis, 5–7: Moderate colitis, 8–9: Severe colitis.

### 2.6. Plasma LPS Determination

Fresh, EDTA-treated blood was centrifuged at 500× *g* at 4 °C for 15 min, to yield plasma. The plasma LPS concentration was assayed using QCL-1000™ Limulus Amebocyte Lysate (LAL, Walkersville, MD, USA) according to the manufacturer’s instructions, where plasma samples were diluted 10-fold and heated for 10 min at 70 °C. The results were quantified at 405 nm using a μQuant microplate reader (Bio-Tek Instruments, Winooski, VT, USA). LPS concentrations are expressed as EU/mL.

### 2.7. Urinary Sucralose Determination

Three mice from each group received 30 mg/mL sucralose by gavage 24 h prior to euthanasia (0.2 mL/25 g BW). Mice were provided food and water ad libitum for the next 6 h, and then placed individually into stainless steel metabolic cages for urine collection, uncontaminated by feces. A sample jar was placed under each cage to collect urine from 6 to 24 h which was then freeze dried and reconstituted in 0.8 mL distilled water for sucralose analysis after conversion of sucralose to its alditol acetate derivatives according to Shaikh and colleagues [16] with minor modifications. Briefly, myo-inositol (9 µL of 10 mg/mL) was added to 100 µL urine sample as internal standard, then 500 µL 20 mg sodium borodeuteride /mL DMSO was added slowly while mixing and heated to 40 °C for 90 min. After cooling, 0.2 mL glacial acetic acid was added slowly while mixing, then 100 µL 1-methylimidazole and 1 mL acetic anhydride were added and the sample stored for 10 min at room temperature, before 4 mL water and 1 mL methylene chloride were added to the mixture and vortexed. The bottom layer was transferred to a new tube. The extraction was repeated three times, and the combined methylene chloride layers washed with 4 mL of water, removed into a fresh tube and dried under a stream of nitrogen. Finally, 0.5 mL acetone was added to the dried residue, vortexed, and the resulting solution analyzed by GC.

Gas chromatography was performed using a 6890N (Agilent Technologies) equipped with a N10149 autosampler and a flame ionization detector. The column used was a 30 m HP-5 capillary column (0.32 mm id, 0.25 µm), with helium as the carrier gas. The analytical program was according to Shaikh and colleagues [16] with some modifications. Briefly, the temperatures of the detector and the injector were 280 °C and 250 °C, respectively; the initial column temperature was 100 °C. After holding for 2 min, the temperature was raised to 180 °C at a rate of 10 °C/min, held for 2 min, then raised to a final temperature of 240 °C at a rate of 4 °C/min, and held for 15 min. The injection volume was 1 µL.

### 2.8. mRNA Expression of Tight Junction Proteins and Pro-Inflammatory Cytokines

Colonic RNA was extracted using an E.Z.N.A.^®^ Total RNA Kit II (Omega Bio-Tek, Norcross, GA, USA) according to the manufacturer’s instructions. Extracted RNA was further purified by Lithium chloride and sodium acetate to remove any remaining DSS, which was reported to interfere with PCR amplification efficiency [17]. RNA was then quantified using a NanoDrop spectrophotometer and converted to cDNA using a High-Capacity cDNA Reverse Transcription kit (Applied Biosystems, Grand Island, NY, USA). Primers for the genes (Table 2) were designed using the online Primer3-BLAST system from NCBI, and were tested for amplification efficiency and specificity with the use of the SYBR Green PCR Master Mix (Applied Biosystems) and an ABI 7900HT Fast Real-Time PCR System (Applied Biosystems) according to the manufacturer’s instructions. All cDNA samples and pairs of primers were submitted to the Roy J. Carver Biotechnology Center (University of Illinois, Urbana, IL, USA) for gene expression profiling using a Fluidigm Dynamic Array and Biomarker HD high-throughput amplification system (Fluidigm, South San Francisco, CA, USA) following 12 cycles of pre-amplification. The copy number for each gene was calculated using standard curves and normalized to the housekeeping gene Hprt1 for each sample.

### 2.9. Protein Expression of Tight Junctions

The colonic mucosal scrapings were homogenized with 0.2 mL RIPA lysis buffer (0.15 M NaCl, 5 mM EDTA, 10 mM Tris-HCl (pH 7.4), 1% Triton X-100, 0.1% SDS, 1% Sodium deoxycholate, 5 mM DTT) with Protease Inhibitor Cocktail (ROCHE, Indianapolis, IN, USA) to prevent protein degradation. Protein concentration was measured using a Bio-Rad protein assay kit (Bio-Rad Laboratories, Indianapolis, IN, USA). Mucosal samples were separated by SDS-PAGE (Bio-Rad) and transferred to a nitrocellulose membrane (GE Health Care, Marlborough, MA, USA) at 60 V for 60 min (for Claudin-1) or 15 V overnight at 4 °C (for ZO-1, which is a larger molecule). After blocking in 5% nonfat dried milk, membranes were incubated with affinity purified rabbit anti-mouse ZO-1 (Invitrogen, Grand Island, NY, USA, 225 kDa, 1:500) or mouse anti-mouse Claudin-1(Life Science, Grand Island, NY, USA, 22 kDa, 1:2500) overnight at 4 °C. After washing with TBST, membranes were incubated with either anti-rabbit secondary antibody (KPL, Milford, MD, USA, 1:5000) or anti-mouse secondary antibody (Santa Cruz, TX, USA, 1:4000), at room temperature for 1 h. Enhanced chemiluminescence of the immunocomplex was evaluated using a detection kit (GE Health Care). Chemiluminescence was visualized using ImageQuant LAS 4000 and IQTL software (GE Health Care). The image was quantified using Quantity One software (Bio-Rad). Band intensities of ZO-1 and Claudin-1 were normalized by the intensity of Ponceau Red staining, used as a loading control [18,19], as the DSS was reported to alter the colonic expression of most commonly used loading control proteins including β-actin and GAPDH in DSS-induced mice [20].

### 2.10. Histology

The formalin-fixed specimens of the middle one-third of the colon from each mouse were trimmed for longitudinal sectioning, with the mucosal surfaces placed face down in the cassette to give full-length profiles of the colonic wall. Following routine processing through graded alcohols, the tissues were embedded in paraffin and sectioned at 4 µm, stained and reviewed in blinded fashion by a board-certified veterinary pathologist (M. A. Wallig). The slides were scored, using a simplified version of a previous scoring system [21]. A severity scale system was used to grade the severity of erosive/ulcerative colitis (EC) and its reparative consequences (persistence/diminution of inflammation; crypt regeneration). A score of 0 indicated no lesions; 1, mild decrease in crypt density and/or mild mucosal edema with mild inflammation, indicative of prior mild damage and/or a repaired lesion with residual inflammation and regenerated/regenerating crypts; 2, focal erosion with mucosal collapse and inflammation affecting <20% of the section, with moderate lymphohistiocytic cellular infiltrates and advanced crypt regeneration along the margins of the erosion; 3, multifocal to locally extensive erosions and mucosal collapse affecting up to 50% of the section, generally with marked lymphohistiocytic infiltration mixed with neutrophils and minimal early marginal crypt regeneration; 4, extensive erosions and mucosal collapse affecting >50% of the mucosa with heavy mixed lymphohistiocytic and neutrophilic infiltrates; 5, ulceration with extension into submucosa and muscularis and accompanying severe neutrophilic inflammation with no marginal regeneration of lost crypts.

### 2.11. Statistical Analysis

The effect of DSS and the interaction between diet and DSS were determined by two-way ANOVA (except for DAI and histological data). Where an interaction was indicated, the effect of DSS was determined by comparing CON diet groups (Water/CON vs. DSS/CON) using Welch’s unequal variance t-test, instead. The effect of diet was determined separately in water and DSS groups, using one-way ANOVA, followed by Tukey’s post hoc test (adjusted for family-wise type I error), where differences were indicated. For DAI and histological data, which are non-continuous quantifications and do not fit normal distribution, the non-parametric ANOVA (Kruskal’s test) was used to determine the effect of diet in the water groups and DSS groups, separately; this was followed by the Wilcoxon rank sum test where differences were indicated. For histological score (EC), Wilcoxon rank sum test with continuity correction was used to determine the effect of DSS in the CON groups (Water/CON vs. DSS/CON). The Pearson’s correlation was used to determine any linear correlation between the EC score and mRNA expression of pro-inflammatory cytokines. For all tests, significance was reported when *p* < 0.05; all tests were carried out using *R*.

## 3. Results

### 3.1. Formation of SF during Diet Hydrolysis In Vitro

**Figure 1:**
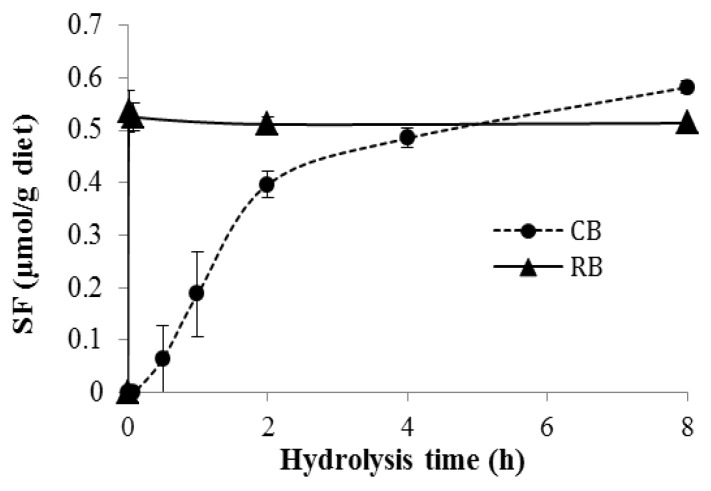
Sulforaphane production by 10% raw broccoli (RB) diet and 10% lightly cooked broccoli (CB) diet after in vitro hydrolysis for 1 min, 5 min, 2 h, and 8 h (RB) and 1 min, 5 min, 30 min, 1 h, 2 h, 4 h, and 8 h (CB), respectively. Data are mean ± SE (*n* = 3–5).

In this study, we compared the efficacy of diets containing a single broccoli preparation, either raw or lightly cooked, for protecting the host from DSS-induced colitis. The lightly cooked broccoli was prepared by microwave heating for 3 min, which retained weak myrosinase activity, as shown in Figure 1. When RB was incubated in water (400 mg diet/1.5 mL water), SF formed rapidly and was complete within 1 min, whereas, when CB was treated similarly, SF started to form as late as 30 min after initiating the incubation and reached a similar maximum SF production but not until 4–8 h later (Figure 1), indicating that CB had much less myrosinase activity, releasing SF at a much slower rate.

### 3.2. Disease Activity Index

**Figure 2:**
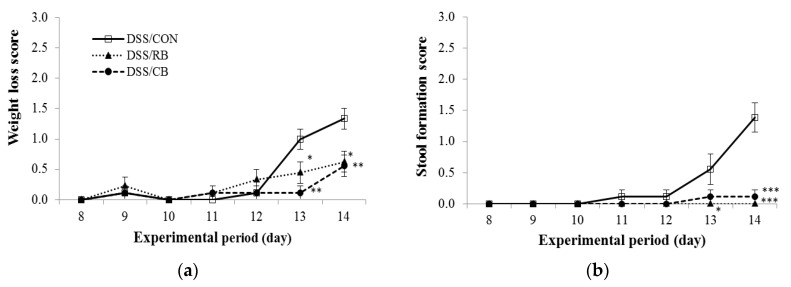

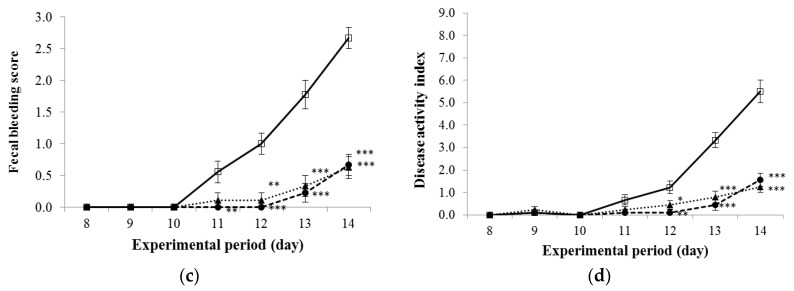
The effect of diet on: (**a**) weight loss; (**b**) stool formation; (**c**) fecal bleeding; and (**d**) the combined disease activity index in dextran sulfate sodium (DSS)-treated mice during DSS treatment (Days 8–14). The diet groups are control (CON, open squares), 10% raw broccoli (RB, filled triangles) and 10% lightly cooked broccoli (CB, filled circles). Data are mean ± SE (*n* = 9, except for RB at Day 14, *n* = 8). *, ** and *** indicate significant difference from DSS/CON (*p* < 0.05, *p* < 0.01 and *p* < 0.001, respectively).

Mice were monitored daily during DSS treatment (Days 8–14), scored for weight loss, stool formation and fecal bleeding. The addition of these scores is the DAI. Weight loss was partly mitigated by RB and CB diets, with 53% and 58% decrease in weight loss, respectively, by the completion of the study, compared to weight loss in mice receiving the CON diet (Figure 2a). The RB and CB diets were more effective at mitigating those symptoms most directly related to the colon, i.e., stool formation and fecal bleeding (Figure 2a,c). By the completion of the study, mice receiving either RB or CB showed a 100% or 92% reduction in the extent of damage to stool formation, respectively, and 77% or 75% reduction in fecal bleeding, respectively, compared to mice receiving the CON diet. 

As a general pattern (Figure 2d), the DAI increased over the period of DSS treatment in CON mice, whereas mice receiving RB and CB exhibited slower increases in scores for damage in all three parameters. The DAIs for RB- and CB-fed mice were significantly different from the DAI for CON mice, starting from Day 12 (five full days of DSS treatment; Figure 2d). After seven days of DSS treatment, RB and CB mice showed 77% and 71% lower DAI, respectively, compared to CON mice. Taken together, these data suggest that feeding mice either RB or CB alleviated the clinical symptoms of colitis, particularly symptoms directly related to colon health.

### 3.3. Colon Length

**Figure 3:**
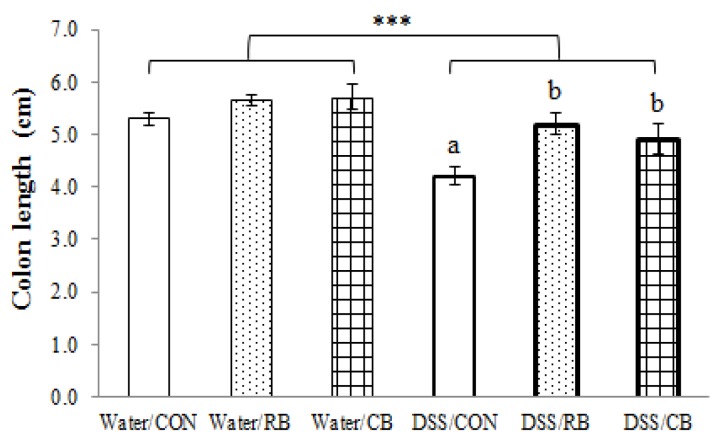
The effect of diet and dextran sulfate sodium (DSS) on colon length. Diet groups are tap water and control (Water/CON), tap water and 10% raw broccoli (Water/RB), tap water and 10% lightly cooked broccoli (Water/CB), DSS and control (DSS/CON), DSS and 10% raw broccoli (DSS/RB), and DSS and 10% lightly cooked broccoli (DSS/CB). Data are mean ± SE (*n* = 6, except for DSS/RB, *n* = 5). *** indicates a significant effect of DSS, *p* < 0.001. The presence of letters indicates a significant effect of diet for Water groups or DSS groups (*p* < 0.05). Values with the same letter are not different (*p* < 0.05).

In mice receiving RB or CB but not DSS (healthy controls), neither diet had any effect on colon length (Figure 3). When given DSS daily for seven consecutive days, the colon length decreased by 21% (DSS-CON vs. Water-CON). In DSS-treated mice, the colon length of those mice receiving RB or CB were 24% and 17% longer than those of CON mice, respectively.

### 3.4. Gut Barrier Permeability

**Figure 4:**
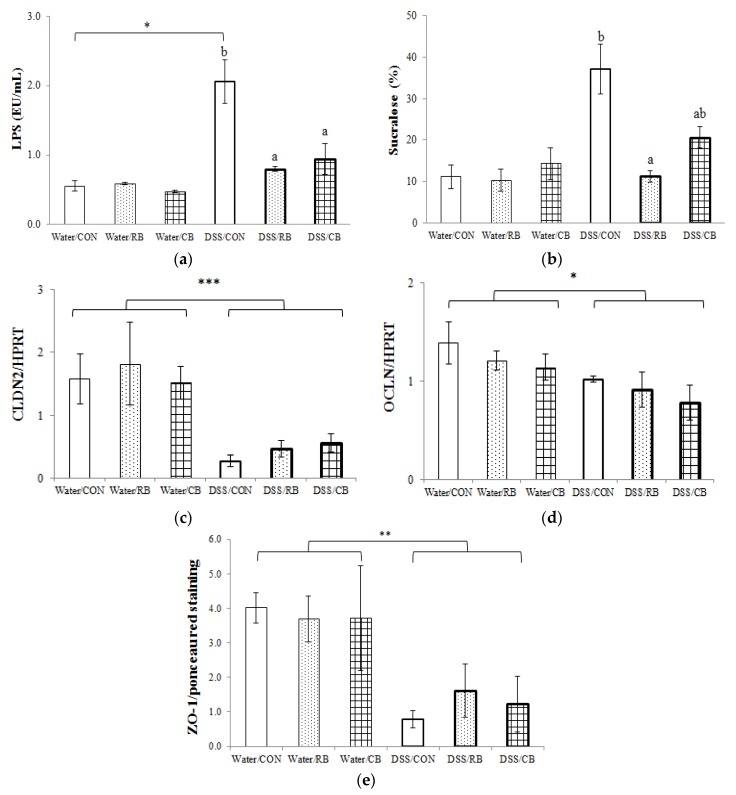
The effect of diet and dextran sulfate sodium (DSS) on gut barrier function: (**a**) plasma lipopolysaccharide (LPS) concentration, *n* = 3, except for DSS/RB (*n* = 4); (**b**) urinary sucralose excretion, *n* = 3, except for DSS/CON and DSS/RB (*n* = 2); (**c**) mRNA expression of CLDN2, normalized by HPRT, *n* = 3, except for DSS/RB (*n* = 4); (**d**) mRNA expression of OCLN, normalized by HPRT, *n* = 3, except for DSS/RB (*n* = 4); and (**e**) protein expression of ZO-1, *n* = 3, except for DSS/CB (*n* = 2). Diet groups are tap water and control (Water/CON), tap water and 10% raw broccoli (Water/RB), tap water and 10% lightly cooked broccoli (Water/CB), DSS and control (DSS/CON), DSS and 10% raw broccoli (DSS/RB), and DSS and 10% lightly cooked broccoli (DSS/CB). Data are mean ± SE. * and *** indicate a significant effect of DSS (*p* < 0.05 and *p* < 0.001, respectively). ** indicates *p* < 0.01; The presence of letters indicates a significant effect of diet within Water groups or DSS groups (*p* < 0.05). Values with the same letter are not different (*p* < 0.05).

Plasma LPS concentration was low in the water groups, regardless of the diet (Figure 4a). When DSS was given to CON mice daily for seven days, the plasma LPS increased four-fold. This increase was far less in plasma LPS from mice receiving either RB or CB (*p* < 0.01 and *p* < 0.05, respectively). 

Similarly, the sucralose level in the urine, which was low in all three water groups, increased three-fold when CON mice were given DSS daily for seven days (Figure 4b). Urinary sucralose was substantially lower following RB (*p* < 0.05) and trended toward lower following CB. 

### 3.5. Tight Junction Expression

Among the selected eight genes involved in the production of tight junction proteins, the mRNA expression of two genes (CLDN2 and OCLN) were significantly decreased by DSS treatment (*p* < 0.001 and *p* < 0.05, respectively; Figure 4c,d). The broccoli diets appeared unable to reverse the effect of DSS. The mRNA expression of the other six genes examined (CLDN1, CLDN3, CLDN4, CLDN5, CLDN8 and TJP1) was not impacted by DSS and the different diets [22].

The expression of two tight junction proteins (Claudin-1 and ZO-1) was also investigated. The DSS treatment decreased expression of ZO-1(*p* < 0.01; Figure 4e), but had no effect on Claudin-1 [22]. Broccoli diets had no impact on levels of either tight junction protein.

### 3.6. mRNA Expression of Pro-Inflammatory Cytokines

**Figure 5:**
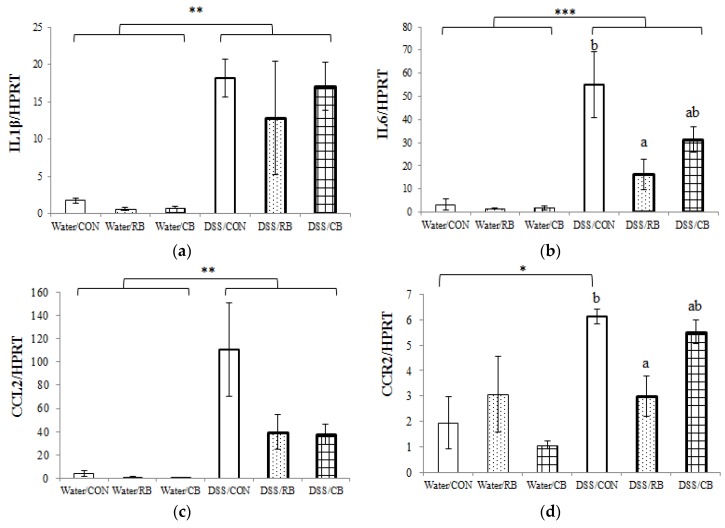

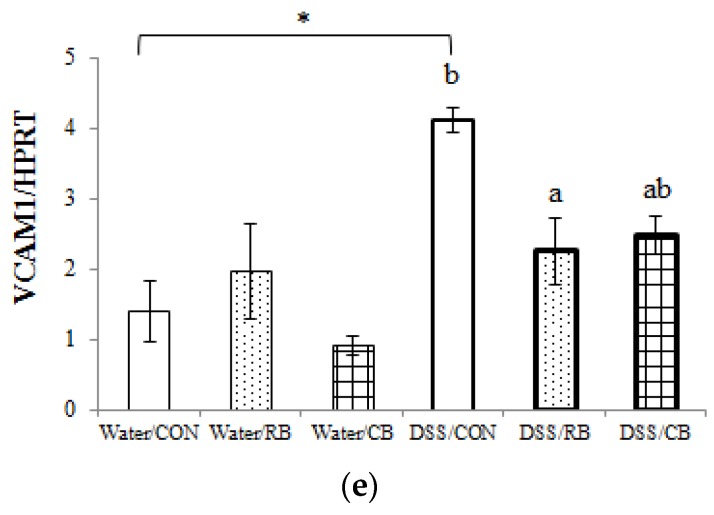
The effect of diet and dextran sulfate sodium (DSS) on mRNA expression of pro-inflammatory cytokines: (**a**) normalized expression of IL1β; (**b**) normalized expression of IL6; (**c**) normalized expression of CCL2; (**d**) normalized expression of CCR2; and (**e**) normalized expression of VCAM-1. Diet groups are tap water and control (Water/CON), tap water and 10% raw broccoli (Water/RB), tap water and 10% lightly cooked broccoli (Water/CB), DSS and control (DSS/CON), DSS and 10% raw broccoli (DSS/RB), and DSS and 10% lightly cooked broccoli (DSS/CB). Data are mean ± SE, *n* = 3, except for DSS/RB (*n* = 4). *, ** and *** indicate significant effect of DSS (*p* < 0.05, *p* < 0.01 and *p* < 0.001, respectively). The presence of letters indicates a significant effect of diet within Water groups or DSS groups (*p* < 0.05). Values with the same letter are not different (*p* < 0.05).

Among the nine selected pro-inflammatory markers, the expressions of five genes (IL1β, IL6, CCL2, CCR2 andVCAM1) were significantly increased by DSS treatment (Figure 5). Notably, the expression of the CCL2 gene was increased 27-fold in DSS mice (DSS/CON vs. Water/CON, Figure 5a). These results suggest that most of the selected pro-inflammatory genes responded to DSS treatment. Looking into the effect of diet in healthy (no DSS) mice, gene expression was not affected by diet in the water groups, suggesting that neither RB nor CB had any effect on the expression of these pro-inflammatory genes in healthy, control mice. However, the diet did influence the expression of three pro-inflammatory genes that had been upregulated by DSS treatment: IL6, CCR2, and VCAM1 (Figure 5b,d,e). Specifically, the expression of IL6 in DSS/RB and DSS/CB mice was 3.4-fold and 1.8-fold lower, respectively, compared to expression in DSS/CON mice. A post-hoc test showed that the expressions of genes IL6, CCR2 and VCAM1, from DSS/RB were different from DSS/CON, although values from tissue of mice receiving DSS/CB were not.

### 3.7. Histology

**Figure 6:**
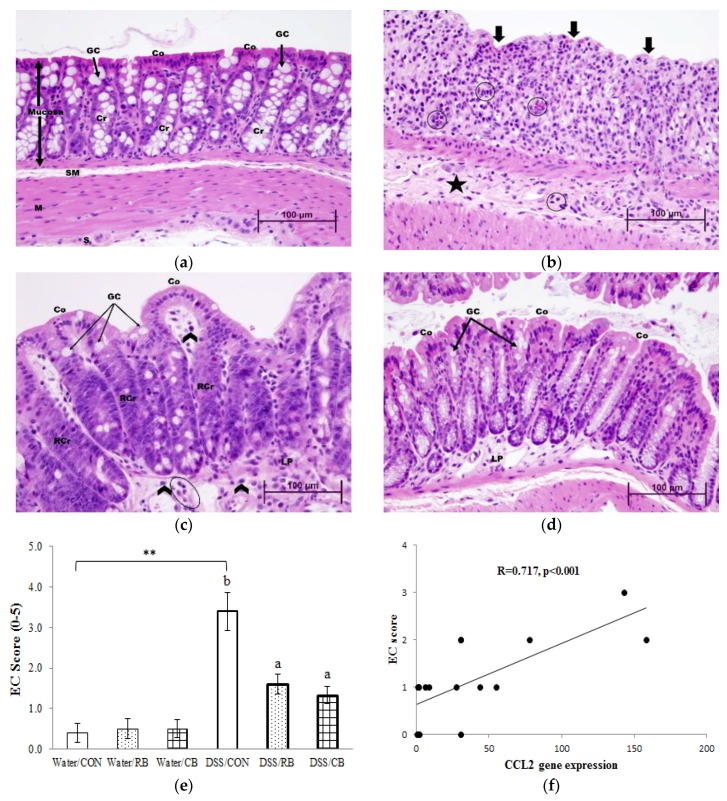
The effect of diet and dextran sulfate sodium (DSS) on histological pathology: (**a**–**d**) representative microscopic images (40× magnification) of colon sections from Water/CON, DSS/CON, DSS/CB and DSS/RB, respectively; (**e**) erosive/ulcerative colitis (EC) scores; and (**f**) the correlation between mRNA expression of CCL2 and EC score. Abbreviations used in microscopic images are: colonocytes (Co), goblet cells (GC), crypts (Cr), regenerating crypts (RCr), lamina propria (LP), submucosa (SM), and muscularis (M). Highlights are the disappearance of colonocytes (bold arrows), neutrophils (encircled), edematous submucosa (star), and residual histiocytes (arrowheads). Diet groups are tap water and control (Water/CON), tap water and 10% raw broccoli (Water/RB), tap water and 10% lightly cooked broccoli (Water/CB), DSS and control (DSS/CON), DSS and 10% raw broccoli (DSS/RB), and DSS and 10% lightly cooked broccoli (DSS/CB). Data are mean ± SE, *n* = 6, except for Water/CON, DSS/CON and DSS/RB (*n* = 5). *, ** and *** indicate a significant effect of DSS (*p* < 0.05, *p* < 0.01 and *p* < 0.001, respectively). The presence of letters indicates a significant effect of diet within Water groups or DSS groups (*p* < 0.05). Values with the same letter are not different (*p* < 0.05).

Representative photography of HE stained colon sections from mice receiving the water control and from the three groups receiving DSS are shown in Figure 6a–d, and the scores for erosive/ulcerative colitis for each group are shown in Figure 6e. Histology from mice receiving Water and RB or Water and CB were not different from water and control diet [22].

Figure 6a shows a typical normal and healthy colon (scored 0), with tall columnar surface colonocytes (Co), abundant goblet cells (GC, arrows), long crypts (Cr) of uniform depth, and an inconspicuous lamina propria (LP), giving the mucosa a densely cellular appearance. Submucosa (SM) is thin and peripheral muscularis (M) is composed almost entirely of densely packed smooth muscle cells. In contrast, Figure 6b, a section of colon from a DSS/CON mouse (scored 4), shows severe mucosal collapse, with the disappearance of colonocytes (bold arrows) and goblet cells. Moreover, throughout the tissue there is intensive leukocyte infiltration (predominantly neutrophils [encircled] and some histiocytes (monocytes which have left the circulation and entered the lamina propria)). The submucosa is edematous (star) as evidenced by its expanded size between mucosa and muscularis. All these changes suggest severe and ongoing inflammation in colons from DSS/CON mice, with no regeneration evident.

In Figure 6c, a colon from a mouse treated with DSS and RB (scored 2), there are elongated, newly regenerating crypts (RCr) and the surface is covered by regenerated colonocytes. A few goblet cells have reappeared. Portions of the lamina propria not fully repopulated by crypts, but there is less edema and only residual neutrophils (circled) and histiocytes (arrowheads), suggesting some recovery from damage. These changes suggest that RB mitigated DSS-induced colonic damage. In Figure 6d, a colon from a mouse given DSS and CB (scored 1), there is even further regeneration to almost normal structures of both crypts and colonocytes are similar to normal colon (Figure 6a). There is very little infiltration by neutrophils and monocytes, and the submucosa is thin with minimal edema. Goblet cells are more numerous, although most are small due to incomplete replenishment of mucin. The lamina propria is almost completely repopulated by crypts. These changes indicate almost complete regeneration. These data suggest that CB can attenuate mucosal damage. In Figure 6e, a substantial difference was observed between the EC score of Water/CON and DSS/CON (*p* < 0.05), suggesting a strong response to DSS. Both RB and CB were able to decrease colitis as scored from histology, compared to tissues from mice receiving only DSS, but only when removing scores for one DSS/CON mouse (#18) that did not respond to DSS. In total, the histologic data confirmed that both RB and CB are effective at mitigating DSS-induced ulceration in colon.

## 4. Discussion

### 4.1. Site of SF Absorption

In this study, we prepared the lightly cooked broccoli by microwaving for 3 min. The broccoli was shown to still retain a low level of myrosinase activity (Figure 1). Heating broccoli for short periods or at low temperatures can increase maximum SF production, by destroying the heat-sensitive epithiospecifier protein (ESP), a myrosinase-associated protein that irreversibly directs hydrolysis away from ITC formation toward inactive nitrile formation [23]. Alternatively, a longer cooking time or higher cooking temperature begins to destroy myrosinase, adversely impacting the efficacy of a broccoli meal [24]. Temperature and timing for ESP inhibition without loss of myrosinase is not readily predicted, since the exact heat sensitivity of ESP and myrosinase may vary with the broccoli variety [10]. Therefore, it is always crucial to determine the remaining myrosinase activity when using cooked broccoli in a study.

In this study, little myrosinase activity remained in CB, but the rate of SF formation was greatly diminished, compared to RB. It takes only around 1 h for food to begin entering the small intestine following ingestion [25]. Therefore, by the time the broccoli diets entered the small intestine (~1 h), only ~30% of GRP from CB was hydrolyzed, whereas ~100% of the GRP from RB would have been hydrolyzed and absorbed, based on the in vitro data in Figure 1. The extent of hydrolysis in CB might have been even less in vivo, where the hydrolysis was in a dynamic situation and might be altered by other factors such as chewing efficiency, and other food components, leaving the majority of GRP (~70%) remaining intact. Because the myrosinase and other proteins are expected to be digested in the small intestine, we propose that the majority of GRP from CB reaches the cecum intact, where microbiota can slowly hydrolyze the GRP to SF. Consequently, compared to RB, where SF is rapidly formed and released during passage through mouth and stomach, the SF from CB is slowly released, but in cecum and colon, the primary site for colitis. 

### 4.2. Protection from DSS Colitis: Comparing CB and RB

We compared the efficacy of RB and CB for their ability to protect the host from DSS-induce colitis. Our results show that CB was essentially as effective as RB in suppressing damage by DSS, for most of the endpoints, such as DAI (Figure 2), colon length (Figure 3), and lesion severity as assessed by histopathology (Figure 6). The exceptions were gut barrier integrity (Figure 4) and mRNA expression of pro-inflammatory biomarkers (Figure 5). 

RB lessened DSS-increased gut permeability, as measured by plasma LPS concentration and urinary sucralose excretion, whereas CB only decreased plasma LPS concentration (Figure 4a,b). Neither broccoli diet induced changes in mRNA or protein expression of tight junction proteins (Figure 4c–e). Three pro-inflammatory biomarkers (IL6, CCR2 and VCAM-1) were identified via mRNA expression as affected by diet. RB reversed DSS-mediated increase in mRNA expression in all three of these inflammatory biomarkers, whereas CB did not. However, CB did have a tendency to decrease the mRNA expression of these genes, especially for VCAM-1 (*p* = 0.054). The failure to detect any difference between the mRNA expression of these biomarkers among CB- and CON-fed DSS mice, might be attributable to the smaller sample size of CB (*n* = 3) compared RB (*n* = 4). Interestingly, whereas there was no difference between CB and CON in DSS mice, there was also no difference between CB and RB for the gene expression data either.

There are several possible explanations for why CB, with far less myrosinase activity, is essentially as effective as RB. Firstly, although the myrosinase activity in CB is far less, CB has an advantage of a topical effect on the gut wall. As we discussed earlier, compared to RB, where SF is rapidly formed and released during mouth or stomach, the SF from CB is slowly released, but right in cecum and colon, the primary site of damage by colitis. Therefore, the SF from CB is more likely to directly impact colitis than SF from RB, where the SF is formed earlier and has to travel via the blood stream to reach the colon. Secondly, although the myrosinase content is far less in CB, we found that the myrosinase-like activity of rat gut microbiota is greatly increased after four or more days of CB intake [26]. Therefore, increased formation of SF by the gut microbiota after 14 days of CB intake might be sufficient to protect the mice from colitis, as effectively as a larger, but systemic, dose of SF from RB.

However, we also keep open to the possibility that the efficacy of RB and CB might be independent of SF in this study. It is possible that other compounds in broccoli such as quercetin sophorosides, alone or in combination with SF, might play a role in mitigating the colitis. Moreover, it is also possible that the combination of other components in broccoli contributed to efficacy, such as through fermentation and/or modification of the gut microbiota, as indicated in a recent mouse study evaluating the impact of different fiber types on DSS-induced colitis [27]. Here, for example, although total fiber content was balanced across diets (Table 1), there was doubtless a small change in fiber composition, thus a possible change in fermentation and butyrate formation, noting that butyrate, similar to sulforaphane, is known to trigger Nrf2 [28]. Therefore, whether the efficacy of RB and CB we observed is only SF-dependent or due to a more general impact of broccoli and the microbiome, waits for further investigation.

### 4.3. Gut Barrier Integrity

Emerging evidence has suggested that gut barrier dysfunction is closely related to the etiology of IBD [11,12]. In this study, gut barrier integrity was assessed by gut permeability (plasma LPS concentration and urinary sucralose excretion) and expression of tight junction proteins. Consistent with the results of gut permeability tests (Figure 4a,b), histologic evaluation of H&E stained colon sections also suggests that RB and CB were able to protect the integrity of the gut barrier, as evidenced by milder damage, enhanced regeneration and less disruption of crypt structure than for CON/DSS mice (Figure 6c,d).

Gut barrier typically consists of colonocyte plasma membrane tight junctions, secreted mucus, and mucosal immune cells [13]. It is reported that the destruction of the tight junctions is one possible mechanism for the breached gut barrier in DSS-treated mice [11] and a variety of phytochemicals, such as naringenin from citrus fruits and berberrubine from berberis, are reported to upregulate tight junction proteins in DSS-treated mice [29,30]. To investigate whether protection by RB and CB against increased gut permeability is through effects on tight junctions, mRNA expression of genes involved in the production of tight junction proteins, as well as protein expression of two tight junction proteins was determined. However, although both RB and CB showed a tendency to reverse the DSS-induced downregulation of mRNA expression of CLDN2 gene and protein expression of ZO-1, there was no significant impact. Interestingly, for the two tight junction proteins examined (Claudin-1 and ZO-1), DSS had no effect on their mRNA expression (CLDN1 or TJP1 genes [22]), and DSS only affected protein expression of ZO-1. These results suggest that further research about the function of different tight junction proteins is needed, but that maybe broccoli protection of the gut barrier is not through maintenance or preservation of tight junctions.

### 4.4. Inflammation

Colonic inflammation in DSS mice was evaluated by both gene expression of pro-inflammatory cytokines and by histology. We investigated gene expression of the three key pro-inflammatory cytokines that mediate inflammation [31]: interleukin-1 (IL-1β), interleukin-6 (IL-6) and tumor necrosis factor alpha (TNFα). Gene expression of IL-1β and IL-6 (Figure 5a,b) were greatly increased by DSS. TNFα is also an important pro-inflammatory cytokine, but we did not observe any increase in gene expression of TNFα by DSS in this study [22], indicating that DSS-induced colitis might not involve TFNα.

Although both broccoli diets decreased IL-6 expression in DSS mice (Figure 5b), IL-1β expression was not impacted by diet. IL-1β is a potent pro-inflammatory cytokine and plays a central role in mediating the inflammatory response [31]. In this study, a significant increase (10-fold) in gene expression of IL-1β was observed in DSS treated mice (*p* < 0.01, Figure 5a), suggesting that DSS-induced colitis involves the IL-1 signaling family. It also indicates a role for gut microbiota (presumably LPS) in DSS colitis, as IL-1β is mainly induced by microbial products [31], consistent with appearance of LPS in plasma in this study (Figure 4a). However, neither CB nor RB was able to reverse this induction. Furthermore, SF formed in the lower gut from CB had no greater impact than SF formed and absorbed in the upper gut from RB. Interestingly, the expression of other genes from the IL-1 family, including TLR2, TLR4 and NF-kB, were not impacted by DSS in this study [22]. 

**Figure 7:**
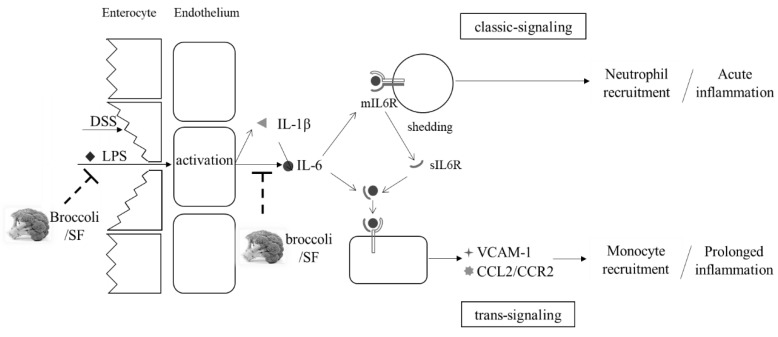
Proposed molecular mechanism of anti-inflammatory property of broccoli is through the IL-6 trans-signaling pathway.

Accumulating evidence suggests that IL-6 is much more than a marker or product of inflammation. It appears to play a pivotal role in both recovery from acute inflammation and the transition from acute inflammation to prolonged/chronic inflammation [32]. In the initial phase of inflammation, the endothelial cells are triggered by microbial products, IL-1β or TNFα, to release IL-6 as well as a series of chemokines [33]. Together, these cytokines recruit neutrophils to the local injury site, the infiltration of which is one important hallmark of acute inflammation [32]. The membrane-bound receptor of IL-6 (mIL6R) found on only a few cell types including hepatocytes and some leukocytes [34] can then be shed as a soluble receptor, sIL6R, freely traveling to combine with IL-6 and complex with gp130, present on multiple cell types. This induces a switch from upregulation of neutrophil-attracting chemokines to upregulation of monocyte-attracting cytokines, including CCL2 (MCP-1)/CCR2, as well as promoting apoptosis of neutrophils and the differentiation of monocytes to form macrophages, hallmarks of chronic inflammation [32]. Typically, acute inflammation is considered beneficial, as it is one of the ways that the body responds to stress, whereas prolonged chronic inflammation may enhance a series of severe diseases. The beneficial acute inflammatory response mediated by IL-6/mIL6R is termed IL-6 classic signaling, whereas the prolonged inflammatory response mediated by the IL-6/sIL6R complex is termed IL-6 trans-signaling [33] (Figure 7). In addition, cell adhesion molecules including VCAM-1 are induced by IL-6 trans-signaling, to increase vascular permeability for monocyte infiltration [33,35,36].

We report here that gene expressions of IL-6 (*p* < 0.001), CCL2 (*p* < 0.01), CCR2 (*p* < 0.01), and VCAM-1 (*p* < 0.01) were increased in DSS mice (Figure 5b–e, respectively), suggesting that chronic inflammation was triggered by IL-6 trans-signaling. Moreover, our data show that RB decreased the upregulation of IL-6, CCR2 and VCAM-1 expression in DSS mice (*p* < 0.05, Figure 5b,d,e, respectively). Notably, as shown in Figure 5c, there was an obvious difference between the mRNA expression of CCL2 of mice fed DSS/CON and DSS/broccoli diets. However, we failed to detect a statistical difference, due to the large variation in the DSS/CON group. Interestingly, if mouse #18 (the DSS non-responder, as described earlier) is removed from the DSS/CON group, both RB and CB showed a significant decrease in the mRNA expression of CCL2 (*p* < 0.01). In addition, a positive correlation between the gene expression of CCL2 and the histological EC score was observed (*R* = 0.717, *p* < 0.001, Figure 6f). As described earlier, the chemokine CCL2 (also referred as monocyte chemoattractant protein 1, MCP1) recruits monocytes, playing an important role in the transition from acute to chronic inflammation. The EC score reflects the degree of ulcerative colitis in the tissue. The positive correlation between CCL2 and EC suggests that the gene expression of CCL2 might be a good marker of colitis. A correlation between the expression of CCL2 and tumor progression was previously reported [37,38], but to the best of our knowledge, the correlation between CCL2 and EC is newly reported here.

We propose that broccoli may be able to halt the transition from acute to chronic inflammation, through the IL-6 trans-signaling pathway. That SF and other brassica ITC inhibit inflammation has been known for a long time, but the exact mechanism is unclear. A recent study proposed a detailed mechanism for the anti-inflammatory effect of Nrf2 [39]. The study found that Nrf2 binds the IL-6 gene to physically interrupt RNA Polymerase II recruitment, effectively inhibiting transcription of IL-6. Moreover, this inhibition involves direct binding of Nrf2 to the gene and is independent of the typical pathway of Nrf2-ARE activation. Given the fact that SF from broccoli is a potent Nrf2 inducer, we propose that the mechanism of broccoli protection may be through direct Nrf2 inhibition of IL-6 expression, leading to suppression of the IL-6 trans-signaling pathway. Consequently, the mRNA expression of genes involved in the IL-6 trans-signaling pathway, IL-6, CCL2/CCR2 and VCAM-1, is inhibited and the transition from acute to chronic inflammation is halted (Figure 7). 

Interestingly, whereas our gene expression data clearly show that the tissue from DSS/CON mice was undergoing a switch from acute inflammation where neutrophils predominate to chronic inflammation where monocytes predominate, the histological data do not support this. Histology showed that neutrophils were still the predominant leukocytes in DSS/CON mice. Nevertheless, the histology data do show that both RB and CB were able to mitigate ulceration in the colon, evidenced by elongated crypts, regenerating colonocytes, and infiltration of fewer neutrophils and monocytes, compared to tissues from DSS/CON mice. This indicates that RB and CB may support a more rapid regeneration of colonocytes and restoration of colonic epithelium, in addition to inhibition of the IL-6 trans-signaling pathway.

## 5. Conclusions

In conclusion, our results clearly show that, even with far less myrosinase activity, CB was essentially as effective as raw broccoli in suppressing damage by DSS, for most of the endpoints, including DAI, colon length, gut barrier integrity as assessed by plasma LPS concentration, and colon lesion severity as assessed by histopathology. Moreover, the pro-inflammatory gene expression data suggest that broccoli protection may be through halting the transition from acute to chronic inflammation through SF-Nrf2 inhibition of the IL-6 trans-signaling pathway. Future studies are needed to test this hypothesis that inhibition of inflammation by SF or broccoli is dependent upon Nrf2 inhibition of IL-6 synthesis and subsequent disruption of IL-6 trans-signaling by Nrf2. 

## Figures and Tables

**Table 1:** Diet composition.

Ingredients (g/100 g Diet)	AIN-93M (CON)	10% Raw Broccoli (RB)	10% Lightly Cooked Broccoli (CB)
Freeze-dried raw broccoli powder	0	10.0	0
Freeze-dried lightly cooked broccoli powder	0.0	0	10.0
Casein	14.0	11.4	11.4
Cornstarch	46.6	44.4	44.4
Maltodextrin	15.5	14.6	14.6
Sucrose	10.0	9.3	9.3
Cellulose	5.0	2.6	2.6
Mineral mix	3.5	2.7	2.7
Vitamin mix	1.0	1.0	1.0
L-cysteine	0.2	0.2	0.2
Choline bitartrate	0.3	0.3	0.3
Soybean oil	4.0	4.0	4.0

**Table 2:** Primer list.

Gene	NCBI Reference Sequence	Forward Primer	Reverse Primer
**Tight Junction**			
CLDN1	NM_016674.4	TGTGGATGGCTGTCATTGGGG	ATTCATACCTGGCATTGATGGGGG
CLDN2	NM_016675.4	ACGGCTCCGTTTTCTAGATGCC	CGTTTGGCTGCTGCTCTTGC
CLDN3	NM_009902.4	AGCCCTCATCGTGGTGTCCA	GGCCGTCTCGTCTTGTACGC
CLDN4	NM_009903.2	GAGCCGTGTTCATCGTGGCA	CCCAGCCGACGTAAAGCGAG
CLDN5	NM_013805.4	GTGCGTGGTGCAGAGTACCG	GAGCGCCGGTCAAGGTAACA
CLDN8	NM_018778.3	ATGCACGGGGGACGATGAGA	TGAGCACAACCAAGCCGGTG
OCLN	NM_008756.2	ACGGTCCTCCTGGCTCAGTT	GATAAGCGAACCTTGGCGGC
TJP1	NM_001163574.1	TGTTTATGCGGACGGTGGCG	TCCATTGCTGTGCTCTTAGCGG
**Inflammation**			
CCL2	NM_011333.3	TTAAAAACCTGGATCGGAACCAA	GCATTAGCTTCAGATTTACGGGT
CCR2	NM_009915.2	ATAAAGGAGCCATACCTGTAAATGC	CATGTGGTGAATCCAATGCCCT
IL1B	NM_008361.4	TGCCACCTTTTGACAGTGATGAGA	TGTTGATGTGCTGCTGCGAGA
IL6	NM_031168.2	TAGTCCTTCCTACCCCAATTTCC	TTGGTCCTTAGCCACTCCTTC
TLR2	NM_011905.3	AGGAGGTGCGGACTGTTTCCT	ATTTGACGCTTTGTCTGAGGTTTCG
TLR4	NM_021297.3	TCCCTGCATAGAGGTAGTTCCTA	TTCAAGGGGTTGAAGCTCAGA
TNF	NM_001278601.1	TCGGTCCCCAAAGGGATGAGA	GGTGGTTTGTGAGTGTGAGGGT
VCAM1	NM_011693.3	ACGTGGACATCTACTCTTTCCCCA	CTTGACCGTGACCGGCTTCC
NFKB1	NM_008689.2	CTGCCATGTCTGCTGCTGCT	CGTGGGCATCACCCTCCAGA
**Housekeeping**			
HPRT	NM_013556.2	TCCCAGCGTCGTGATTAGCG	TCGAGCAAGTCTTTCAGTCCTGT
